# BSA Binding and Aggregate Formation of a Synthetic Amino Acid with Potential for Promoting Fibroblast Proliferation: An In Silico, CD Spectroscopic, DLS, and Cellular Study

**DOI:** 10.3390/biom14050579

**Published:** 2024-05-14

**Authors:** Hayarpi Simonyan, Rosanna Palumbo, Satenik Petrosyan, Anna Mkrtchyan, Armen Galstyan, Ashot Saghyan, Pasqualina Liana Scognamiglio, Caterina Vicidomini, Marta Fik-Jaskólka, Giovanni N. Roviello

**Affiliations:** 1 Institute of Pharmacy, Yerevan State University, 1 Alex Manoogian Str., Yerevan 0025, Armenia; 2 Institute of Biostructures and Bioimaging, Italian National Council for Research (IBB-CNR), Area di Ricerca Site and Headquarters, Via Pietro Castellino 111, 80131 Naples, Italy; 3 Department of Chemistry, Yerevan State University, 1 Alex Manoogian Str., Yerevan 0025, Armenia; 4 Department of Sciences, University of Basilicata, Via dell’Ateneo Lucano 10, 85100 Potenza, Italy; 5 Faculty of Chemistry, Adam Mickiewicz University in Poznań, Uniwersytetu Poznańskiego 8, 61-614 Poznań, Poland

**Keywords:** in silico, protein binding, self-assembling system, synthetic amino acid, fibroblast growth enhancement, tissue regeneration

## Abstract

This study presents the chemical synthesis, purification, and characterization of a novel non-natural synthetic amino acid. The compound was synthesized in solution, purified, and characterized using NMR spectroscopy, polarimetry, and melting point determination. Dynamic Light Scattering (DLS) analysis demonstrated its ability to form aggregates with an average size of 391 nm, extending to the low micrometric size range. Furthermore, cellular biological assays revealed its ability to enhance fibroblast cell growth, highlighting its potential for tissue regenerative applications. Circular dichroism (CD) spectroscopy showed the ability of the synthetic amino acid to bind serum albumins (using bovine serum albumin (BSA) as a model), and CD deconvolution provided insights into the changes in the secondary structures of BSA upon interaction with the amino acid ligand. Additionally, molecular docking using HDOCK software elucidated the most likely binding mode of the ligand inside the BSA structure. We also performed in silico oligomerization of the synthetic compound in order to obtain a model of aggregate to investigate computationally. In more detail, the dimer formation achieved by molecular self-docking showed two distinct poses, corresponding to the lowest and comparable energies, with one pose exhibiting a quasi-coplanar arrangement characterized by a close alignment of two aromatic rings from the synthetic amino acids within the dimer, suggesting the presence of π-π stacking interactions. In contrast, the second pose displayed a non-coplanar configuration, with the aromatic rings oriented in a staggered arrangement, indicating distinct modes of interaction. Both poses were further utilized in the self-docking procedure. Notably, iterative molecular docking of amino acid structures resulted in the formation of higher-order aggregates, with a model of a 512-mer aggregate obtained through self-docking procedures. This model of aggregate presented a cavity capable of hosting therapeutic cargoes and biomolecules, rendering it a potential scaffold for cell adhesion and growth in tissue regenerative applications. Overall, our findings highlight the potential of this synthetic amino acid for tissue regenerative therapeutics and provide valuable insights into its molecular interactions and aggregation behavior.

## 1. Introduction

The continuous quest for novel pharmacologically active agents and the refinement of existing drugs represent enduring challenges in the landscape of pharmaceutical research and development. Within this dynamic arena, amino acids emerge as indispensable molecular scaffolds owing to their intrinsic chemical versatility and pivotal biological roles. Both proteinogenic and non-proteinogenic amino acids serve as foundational building blocks for drug design and discovery, offering a myriad of opportunities to engineer compounds with tailored pharmacological properties [1,2,3,4,5,6,7,8,9,10,11]. A particularly intriguing avenue in drug development lies in the strategic incorporation of heterocyclic ring systems into amino acid structures. Among these heterocycles, oxadiazoles stand out for their remarkable versatility and broad applicability across diverse scientific domains, including medicinal chemistry, pesticide development, polymer science, and material engineering [12]. In the realm of drug discovery and development, several compounds incorporating an oxadiazole component are undergoing advanced clinical trials, such as Zibotentan, which exhibits anticancer properties [13,14]. Of the isomeric forms, particular attention is focused on 1,3,4-oxadiazoles [15] due to their significant relevance across diverse scientific domains, including the pharmaceutical industry, drug discovery efforts, the production of scintillating materials, and the dyestuff industry [16]. The inherent structural diversity and synthetic accessibility of oxadiazoles make them attractive candidates for the design of novel therapeutic agents with enhanced biological activities and improved pharmacokinetic profiles. In the context of tissue regeneration, the quest for innovative strategies to promote healing and tissue repair remains a pressing priority in modern medicine. Tissue regeneration encompasses a complex series of biological processes aimed at restoring the structure and function of damaged or diseased tissues, spanning from wound healing to the regeneration of complex organs. Central to these processes is the pivotal role of fibroblasts, key cellular players involved in orchestrating tissue repair through the synthesis and remodeling of the extracellular matrix (ECM) [17,18]. One promising approach to augmenting tissue regeneration involves the development of biomaterial scaffolds that mimic the native tissue microenvironment and provide a conducive milieu for cellular adhesion, proliferation, and differentiation. In this context, the formation of aggregates of growth-enhancing molecules, such as amino acids bearing heterocyclic functionalities, can serve as a scaffold matrix with a porous structure. This scaffold not only offers physical support but also presents binding sites or adhesive motifs that facilitate robust interactions with fibroblasts, thereby promoting their attachment, spreading, and migration [19,20,21,22]. Moreover, the scaffold matrix laden with growth-enhancing molecules can act as a reservoir for growth factors and signaling molecules, which exert pleiotropic effects on fibroblast behavior, including proliferation, differentiation, and ECM synthesis. Through controlled release mechanisms, these bioactive molecules ensure sustained exposure of fibroblasts to trophic signals, thereby fostering long-term tissue regeneration and remodeling [19,20,21,22]. By harnessing the synergistic interplay between biomaterial scaffolds and bioactive molecules, including amino acid derivatives incorporating heterocyclic functionalities, researchers aim to develop next-generation regenerative therapies capable of harnessing the innate regenerative capacity of the body. Through meticulous characterization and optimization of these biomimetic systems, coupled with mechanistic insights into their mode of action, the ultimate goal is to pave the way for transformative advances in tissue engineering and regenerative medicine. 

Non-natural amino acids present a novel avenue in the realm of tissue regeneration, offering a range of functionalities that can be harnessed for biomedical applications. One notable example is the development of hydrogels using low-molecular-weight gelators (LMW), such as Fm-Dap (Fm), which incorporate non-proteinogenic amino acids such as 2,3-diaminopropionic acid (Dap). These hydrogels exhibit remarkable versatility, forming robust three-dimensional networks that can withstand a wide range of pH conditions. Moreover, their mechanical strength and thermal stability vary according to pH, with enhanced stability observed under acidic conditions [23]. Notably, Fm-Dap (Fm) hydrogels display intriguing properties such as thixotropy and self-healing, making them suitable for various biomedical applications. Morphological analysis reveals the formation of fibrillar structures within the hydrogel matrix, indicative of strong intermolecular interactions such as aromatic π-π stacking and hydrogen bonding. Furthermore, these hydrogels demonstrate biocompatibility, promoting cell viability and proliferation at physiological pH levels [23]. In addition to their structural and biocompatible properties, Fm-Dap (Fm) hydrogels have shown promise in drug encapsulation and release. Co-incubation with compounds such as vitamin B12 results in the sustained release of the drug over time, further expanding their utility in controlled drug delivery systems [23]. Non-natural amino acid hydrotropes, known for their remarkable water-holding capacity and efficacy in skin moisturization [24], hold promise for applications beyond skincare, particularly in tissue regeneration. Their ability to retain moisture and promote hydration is crucial in creating a conducive environment for cell proliferation and tissue repair. By enhancing moisture uptake into the skin, these hydrotropes may facilitate the hydration of damaged or dry tissues, supporting the regeneration process [25]. As for the broader spectrum of tissue regeneration applications involving non-natural amino acids, the modification of sodium montmorillonite (Na-MMT) clay with unnatural amino acids emerges as a promising strategy for crafting intercalated clay structures with potential applications in bone biomaterials. Previous studies on polymer-clay nanocomposites (PCNs) have emphasized the pivotal role of selecting appropriate modifiers to enhance material properties. Previous investigation unveiled a notable increase in the d-spacing of Na-MMT clay following modification with three distinct unnatural amino acids [26]. Cell culture experiments provided compelling evidence of the biocompatibility of Na-MMT clay modified with the non-natural amino acids. Additionally, films comprising chitosan/polygalacturonic acid/hydroxyapatite, incorporating the modified clay, also demonstrated biocompatibility. These findings underscore the potential utility of synthetic amino acid-modified Na-MMT clay in biomedical contexts, particularly in the realm of bone biomaterials [26]. In this context, the current study endeavors to elucidate the potential of a novel non-natural amino acid derivative as a multifunctional regulator of tissue regeneration. By leveraging an integrative approach encompassing chemical synthesis, physicochemical characterization, cellular assays, and molecular modeling, we seek to unravel the intricate interplay between biomaterial scaffolds, bioactive molecules, and cellular processes underlying tissue regeneration. Through these concerted efforts, we aspire to unlock new therapeutic modalities that harness the regenerative potential of the body, ultimately ushering in a new era of personalized regenerative medicine.

## 2. Materials and Methods

The silica gel L-40/100 was purchased from “Merck” (Germany), Ni(NO_3_)_2_•6H_2_O, K_2_CO_3_, (CH_2_O)_n_, CHCl_3_, (CH_3_CO)_2_O, CH_3_COOH, CH_3_COCH_3_, CH_3_CN, CH_3_OH, Na_2_CO_3_, NH_4_OH, HCl, KOH, C_2_H_5_OH, DMF and 2-aminobenzophenone from “Aldrich” (St. Louis, MA, USA). The nucleophilic reagent was synthesized in the Department of Organic Chemistry at YSU. All solvents used were freshly distilled. The ^1^H NMR spectra were recorded on a Mercury-300 Varian (300 MHz, Varian, Palo Alto, CA, USA), while the optical rotation was measured on a Perkin Elmer-341 polarimeter (Perkin Elmer, Waltham, MA, USA). The melting point values were determined using a Melting point Stuart SMP30 apparatus (Bibby Scientific, Stone, UK). Sample preparation: 1 mg of amino acid was dissolved in 10 mL of 0.1% formic acid aqueous solution. The sample solution was filtered through a 0.22-μm syringe filter PTFE 100. ESI MS Analysis: Sample analysis utilized a Prominence I LC-2030C 3D Plus instrument from Shimadzu, Kyoto, Japan. The mobile phase comprised a mixture of 0.1% formic acid aqueous solution (20%) and methanol (80%). A flow rate of 0.2 mL/min was maintained, with the column temperature set at 30 °C. The injection volume stood at 0.1 μL. Detection of the sample employed a basic quadrupole MS system (LC-MS-2020, Shimadzu, Japan) operating in positive ionization mode via electrospray ionization (ESI). Nitrogen gas served as both the nebulizing and drying agent, with the interface temperature, heat block, and DL temperature set at 350 °C, 200 °C, and 250 °C, respectively. Data acquisition and processing were performed using Shimadzu’s LabSolutions software (version 5.99 SP2, Shimadzu, Kyoto, Japan). The chromatogram outlines the monitored *m*/*z* values of the target sample under experimental conditions. IR characterization: The infrared analysis made use of an ATR (Attenuated Total Reflection) accessory, which allowed us to conduct a direct examination of a powder sample using the IRTracer-100 instrument (Shimadzu, Kyoto, Japan).

Synthesis of (S)-2-amino-3-(5-phenyl-2-thioxo-1,3,4-oxadiazol-3(2H)-yl)propanoic acid (**5**). The general synthesis method for complex 3 involved adding 3.6 g (0.007 mol) of 1 in 30 mL of DMF to which 0.78 g (0.014 mol) of KOH and 3.56 g (0.02 mol) of nucleophile 2 were added. The reaction mixture was stirred at room temperature or 50 °C under an argon atmosphere. The reaction progress was monitored by TLC (SiO_2_, CH_3_COCH_3_/CHCl_3_, 1:3) to observe the disappearance of the initial complex 1. Upon completion, the mixture was neutralized with CH_3_COOH and poured into stirred distilled water. After 15 min, the mixture was filtered and dried in air. The decomposition of diastereomeric complex 3 and isolation of the target β-substituted α-amino acid 5 were carried out according to a previously developed procedure [27]. The (S)-2-amino-3-(5-phenyl-2-thioxo-1,3,4-oxadiazol-3(2H)-yl)propanoic acid 5 (52% yield) exhibited a melting point of 199–201 °C; enantiomeric excess (TLC): higher than 98%; [α]_D_^20^ = +5.3 (C = 0.5; 6N HCl). Found (%)C, 49.30; H, 4.25; N, 15.92; O, 18.15; S, 12.03; Calc for C_11_H_11_N_3_O_3_S (%) C, 49.80; H, 4.18; N, 15.84; O, 18.09; S, 12.09. ESI MS (*m*/*z*): 265.95 (found) 266.29 (expected for [C_11_H_11_N_3_O_3_S]+H^+^). ATR-IR, ν, cm^−1^: 3259.70 (NH in NH_2_, NH_3_^+^); 3167.12 (OH, NH in NH_2_, NH_3_^+^); 3062.96 (C-Harom); 2978.09 (C-H); 1732.08 (C=O); 1705.07 (C=O); 1643.35 (C=N); 1600.92 (C=Carom); 1577.77; 1512.19; 1477.47; 1438.90; 1408.04; 1342.46; 1307.74; 1280.73; 1249.87; 1226.73; 1184.29 (C=S); 1095.57; 1072.42; 1022.27; 999.13; 929.69; 902.69; 794.67; 771.53; 721.38; 702.09; 686.66; 655.80; 613.36; 559.36; 520.78; 408.91; 960.38; 943.98; 937.72; 925.66; 897.22; 859.13; 802.24; 756.44; 714.01; 657.13; 638.80; 622.41; 595.41; 580.95; 554.43; 525.51; 513.45; 487.42; 474.88; 438.73; 410.28.

^1^H-NMR (DMSO/CCl_4_ 1/3 + CF_3_COOD) δ, p.p.m.: 3.88 dd (1H, J = 9.0, 5.6, CH); 4.13 dd (1H, J = 10.6, 9.0, CH2); 4.48 ddd (1H, J = 10.6, 5.6, 0.7 CH2); 7.47–7.53 m (2H, H-3,3′ Ph); 7.56–7.62 m (1H, H-4 Ph); 7.86–7.90 m (2H, H-2.2′ Ph); 9.13 br (1H, NH2); 10.72 s (1H, NH2); 13.10 br (1H, COOH). ^13^C-NMR (DMSO) 52.3 (CH3); 53.8 (CH); 127.4 (C-2,2′ Ph); 128.4 (C-3,3′ Ph); 132.0 (C-4 Ph); 132.2; 165.1; 171.7; 184.33.

### 2.1. Dynamic Light Scattering

Dynamic light scattering (DLS) experiments were conducted using a Zetasizer Nano ZS instrument (Malvern Instruments, Malvern, UK), employing 12 mm square polystyrene cuvettes (DTS0012, Malvern Instruments). The solution containing compound **5** was formulated to a concentration of 140 μM in 1X PBS (pH 7.4) at room temperature. Each analysis was repeated three times, employing a scattering angle of 173° and an equilibration period of 60 s.

### 2.2. CD Binding Studies

Circular dichroism (CD) spectra were recorded using a Jasco J-715 spectropolarimeter (Jasco, Tokyo, Japan) equipped with a Peltier PTC-423S/15 accessory. The experiments were conducted at a temperature of 22 °C in phosphate-buffered saline (PBS) with a pH of 7.4. The stock solution of compound **5** was prepared at a concentration of 4.6 mM in ethanol. For the experiments, 1.25 nmol of bovine serum albumin (BSA) (Merck, Darmstadt, Germany) and 37.5 nmol of compound **5** were used. The volume after mixing was 2 × 0.8 mL, and the optical path length (b) was 2 × 0.4375 cm, using a Hellma-238-QS tandem quartz cell (Hellma Italia S.r.l., Milano, Italy). Each spectrum was an average of three scans.

### 2.3. CD Deconvolution

To analyze the circular dichroism spectra, CD3 software (available at http://lucianoabriata.altervista.org/jsinscience/cd/cd3.html, accessed on 25 March 2024 [28]) was utilized, where CD (mdeg) and wavelength (nm) data were inputted. Specifically, only data associated with positive coefficient values were chosen for the analysis of protein structure, opting for the ‘Fit alpha beta coil’ option.

### 2.4. UV Spectroscopy

UV spectra were recorded using a JASCO J-815 CD spectropolarimeter (Jasco Inc., Easton, MD, USA). For this study, solutions of compound **5** were prepared in 25 mM sodium phosphate buffer (pH 7.4) across a concentration range of 0.016–0.5 mM. Background correction was applied to all spectra by subtracting the appropriate blank. UV experiments were conducted in duplicate.

### 2.5. Molecular Docking

The molecular docking investigations described in this study made use of the HDOCK software (v1.1, Huazhong University of Science and Technology, China) [29,30]. HDOCK is a widely utilized computational tool for conducting docking simulations involving macromolecules, including proteins [31,32]. Through the utilization of ITScore-PP, an iterative knowledge-based scoring function, HDOCK ranks the top poses generated during docking runs, typically providing the top 1–10 poses. The resulting HDOCK scores are dimensionless and serve as indicators of binding affinities [33]. More negative HDOCK scores are indicative of higher binding affinities, facilitating the comparison of different ligands’ affinities for the same biomolecular target [33]. The aggregate model was derived through blind self-docking simulations initiated from compound **5**, employing default parameters. The resulting complexes were subsequently analyzed using Discovery Studio software (BIOVIA, Dassault Systèmes, San Diego, CA, USA) [34], which was also utilized for examining the intermolecular interactions within the complexes. Through these procedures, we obtained the (5)n aggregate in silico, encompassing various values of n ranging from 0 to 512. The BSA–ligand interaction 3D diagram reported in this paper was obtained using PLIP (Protein–Ligand Interaction Profiler, https://plip-tool.biotec.tu-dresden.de/, accessed on 25 March 2024).

### 2.6. Cellular Studies

Normal dermal fibroblast (HDF) cells were maintained in DMEM supplemented with 2% FBS, 1 μg/mL hydrocortisone, 10 ng/mL human epidermal growth factor, 3 ng/mL basic fibroblast growth factor, and 10 μg/mL heparin. All media and supplements were purchased from Thermo Fisher Scientific (Milan, Italy). For cytotoxicity assays, 5 × 10^3^ normal cells were seeded in 50 μL of medium per well in 96-well flat bottom microplates and incubated overnight at 37 °C in a 5% CO_2_-humidified atmosphere to allow cell adhesion. The culture medium was then replaced with 100 μL of fresh growth medium containing different concentrations of compounds and incubated for 24 h. Compound **5** was dissolved in water at a concentration of 8.75 mM. Cell viability was assessed using the crystal violet assay. The amount of dye absorbed by the cells was measured using a plate reader (Multiskan Fc 10094, Thermo, Waltham, MA, USA) at 595 nm. Data were expressed as the percentage of proliferating cells relative to the control (vehicle-treated cells) and represented as means ± standard deviation (SD) of two independent experiments conducted in triplicate.

## 3. Results

**Scheme 1:**
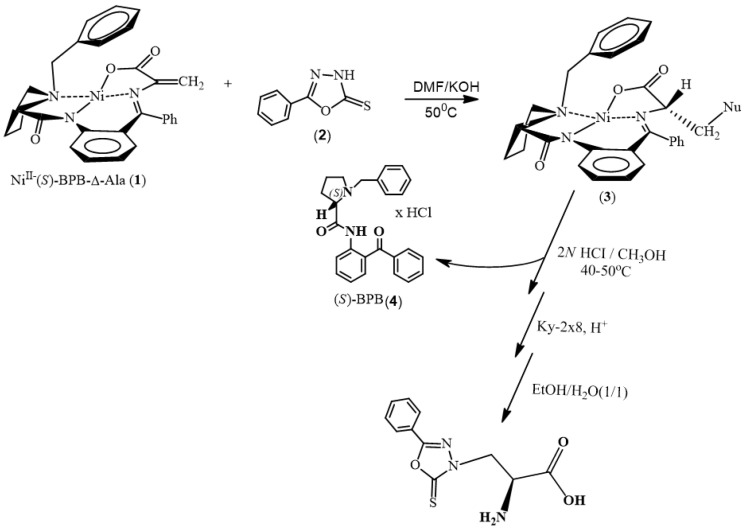
Synthetic route to compound **5**, depicting the stepwise procedure starting from the chiral NiII complex of Schiff’s base of dehydroalanine (NiII-(S)-BPB-Δ-Ala) (**1**) with the chiral auxiliary (S)-2-N-(N’-benzylprolyl) aminobenzophenone ((S)-BPB) (**4**), followed by the addition of substituted 1,3,4-oxadiazole (**2**) and subsequent isolation and recovery steps to obtain the desired α-amino acid **5** from the main diastereomeric complex **3**.

The synthesis of non-natural amino acids through metal-catalyzed chemical synthesis has emerged as a significant area of research in organic chemistry [35,36,37,38]. Leveraging the versatility of transition metal catalysts, researchers have devised efficient strategies for the preparation of diverse amino acid derivatives with tailored structures and functionalities. One notable approach involves the utilization of chiral metal complexes, such as the chiral NiII complex described herein, for the asymmetric synthesis of amino acids. More in detail, the chiral NiII complex of Schiff’s base of dehydroalanine (NiII-(S)-BPB-Δ-Ala) (1) with the chiral auxiliary (S)-2-N-(N’-benzylprolyl) aminobenzophenone ((S)-BPB) (4) was synthesized following a previously reported procedure [39]. Subsequently, the addition of substituted 1,3,4-oxadiazole (2) to the chiral dehydroalanine complex NiII-(S)-BPB-Δ-Ala (1), conducted in dimethylformamide in the presence of KOH, yielded complex 3 (Scheme 1) with good to very good diastereoselectivities. The desired α-amino acid 5 was isolated from the main diastereomeric complex **3** following acidic hydrolysis and ion-exchange demineralization (Scheme 1). The chiral auxiliary (S)-BPB (4) was recovered in a quantitative yield (>95%) without any loss of optical purity and could be reused. Amino acid 5 was subsequently obtained after crystallization from water:ethanol (1:1) with a yield of 52% and characterized by NMR (Figures S1 and S2).

### Self-Assembly and Biological Properties of the Non-Natural Amino Acid ***5***

**Figure 1:**
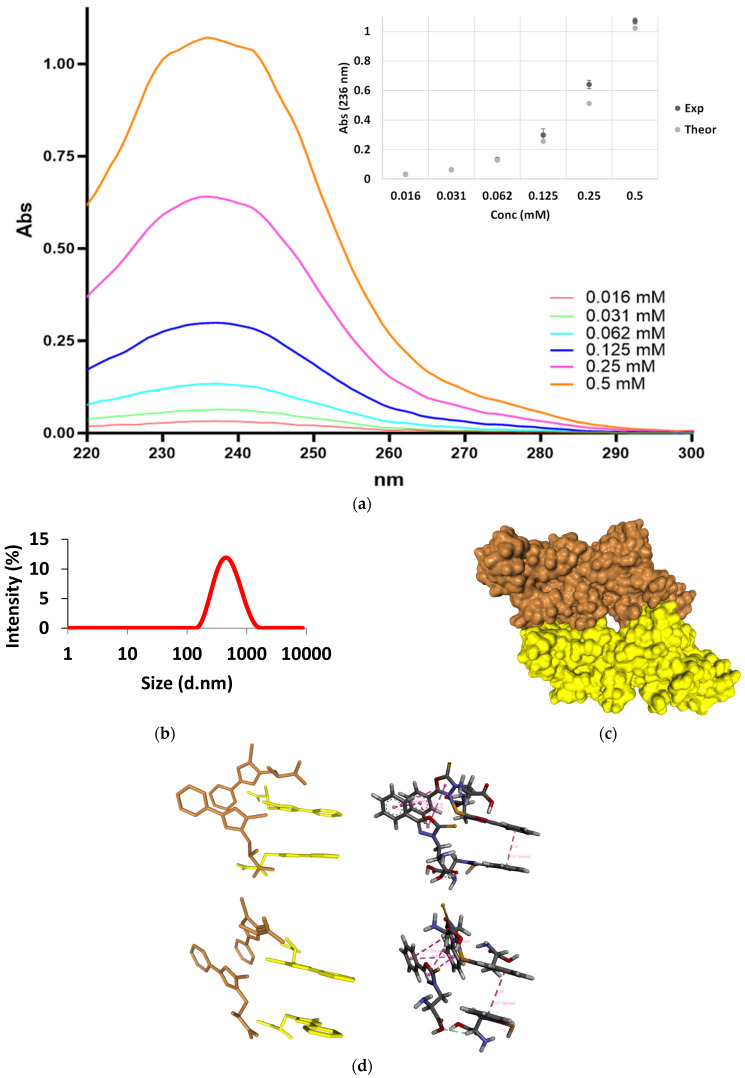
Plot of UV Absorbance against concentration of compound aggregation (**a**). DLS analysis of compound **5** in PBS 1X pH 7.4 at room temperature (**b**). Model of an aggregate formed by 512 molecules of compound **5**. Note the presence of a cavity within its interior (**c**). Two 3D pose views of the tetrameric complex formed by the coplanar dimer (yellow) and the ring-staggered dimer (brown), with interaction types highlighted in purple (π-π stacking) and grey (carbon–hydrogen bond). Note how the carbon–hydrogen bond holds together the two dimeric structures into the tetramer and how the stacking also occurs in the staggered dimer (**d**).

To explore the potential of this non-natural amino acid to form supramolecular networks, similar to examples reported in the literature [40], we conducted specific experimental and computational studies. More in detail, we performed UV and Dynamic Light Scattering experiments to assess its capability in this regard. These studies aimed to provide insights into whether the amino acid could form aggregates and to determine the size of the molecular systems it could generate. Figure 1a displays the outcomes of UV experiments conducted on buffered solutions of compound **5** at various concentrations in phosphate buffer (pH = 7.4). The deviation from linearity observed in Figure 1a suggests the aggregation of the investigated compound. Figure 1b shows the results of the DLS analysis conducted on compound **5** in phosphate-buffered saline (PBS) solution with a pH of 7.4. The measurements, performed at room temperature, provided insights into the size distribution of particles in the sample based on their hydrodynamic diameter (d.nm). The DLS data suggests that the dominant size in the distribution is approximately 391 nm, with a polydispersity index (PdI) of 0.262. This polydispersity index indicates a relatively narrow size distribution, suggesting that the particles in the sample have a similar size range. This can be interpreted as a homogenous population of particles with minimal variation in size. The significant representation of particles around 391 nm suggests that this particular size is prevalent in the sample. Additionally, we employed HDOCK software [41,42,43] to conduct in silico oligomerization of the synthetic compound, aiming to generate a model of aggregate for computational investigation (Figure 1c). The self-assembly of a nucleobase-bearing amino acid derivative was recently studied by self-docking simulations [3]. In self-docking simulations, multiple copies of the molecule of interest are employed, and each copy is treated both as a ligand and a receptor. The goal is to predict the most energetically favorable arrangements of these molecules and to understand the forces driving their self-assembly. Self-docking simulations can provide valuable insights into the formation of supramolecular structures, such as protein oligomers, nucleic acid complexes, or nanomaterials. By analyzing the docking poses and energy scores, one can elucidate the key interactions responsible for stabilizing these structures, such as hydrogen bonds, van der Waals forces, and π-π stacking interactions. In the context of the study by Jitaru et al., which explores the self-assembly mechanism of the amidated FEYNF peptide, molecular docking simulations were employed to gain comprehensive insights into the process at the origin of the hydrogelation [44]. The best docked FEYNF@FEYNF dimer complex conformation highlighted intermolecular interactions based on hydrophobic interactions, π-π stacking, and hydrogen bonds. This structural arrangement facilitated the self-assembly of the FEYNF-NH2 peptide into a hydrogelated dendritic architecture, as observed experimentally. This study demonstrates the utility of molecular docking simulations in elucidating the self-assembly mechanism of peptide-based materials, providing valuable insights into their structural organization and potential applications in supramolecular gelation. In the paper by Mosseri et al., molecular docking was utilized to construct a model of a WWgc fiber starting from a WW dipeptide unit [45]. The dipeptide unit was used as both a receptor and a ligand in the Haddock software [46,47,48,49,50] to predict its dimeric form through molecular docking techniques. Haddock generated a set of structures from which the most energetically favorable structure of the best cluster was selected. This structure represented the dimeric arrangement of the WW dipeptide units. The selected dimeric structure was then utilized to develop WWgc conjugates (g and c stand for guanine and cytosine PNA monomeric units), which are essential for the formation of WWgc fibers [45]. Overall, the study demonstrated how molecular docking techniques could be employed to investigate the self-aggregation of amino acid units and to model the formation of higher-order structures such as WWgc fibers. Despite the unconventional use of docking for self-aggregation studies, the results provided insights into the potential structural arrangements and interactions involved in the assembly of these fibers. Specifically, during the formation of dimers via molecular self-docking, we observed two distinct poses (Figure 1d). One pose displayed a quasi-coplanar arrangement (Figure 1d, yellow) characterized by the close alignment of two aromatic rings from the synthetic amino acids within the dimer, suggesting the presence of π-π stacking interactions as confirmed by analysis with the Discovery Studio software (Figure 1d, right). In contrast, the second pose (Figure 1d, brown) exhibited a non-coplanar configuration, with the aromatic rings oriented in a staggered arrangement, indicating different modes of interaction and potential variations in stability and binding affinity. Both poses were further utilized in the self-docking procedure.

**Figure 2:**
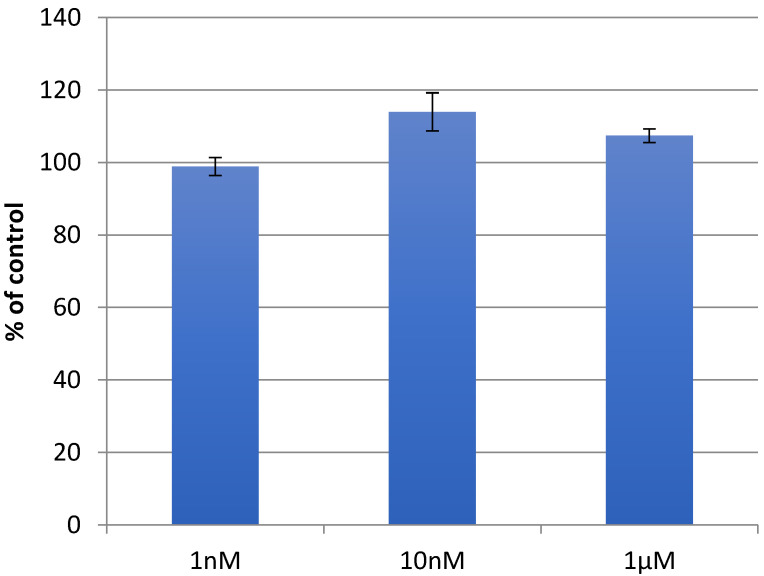
Growth of HDF fibroblasts (%) with 1 nM, 10 nM, and 1 μM concentrations of compound **5**. Control corresponds to vehicle-treated cells.

Remarkably, iterative molecular docking of amino acid structures using HDOCK resulted in the formation of higher-order aggregates, culminating in a model of a 512 mer aggregate obtained through self-docking procedures (Figure 1c). This aggregate model featured a cavity (Figure 1c) capable of hosting therapeutic cargoes or biomolecules, rendering it a potential scaffold for cell adhesion and growth in tissue regenerative applications. To assess whether scaffolds resembling those formed by the aggregation of compound **5** could induce enhanced fibroblast growth, similar to other analogous literature examples, we conducted cellular studies (Figure 2). We observed that at a concentration of 10 nM, compound **5** was capable of promoting fibroblast growth (Figure 2), and this effect persisted even at higher concentrations. These findings suggest that the aggregates of the non-natural amino acid support fibroblast growth, thus offering potential utility in regenerative approaches.

**Figure 3:**
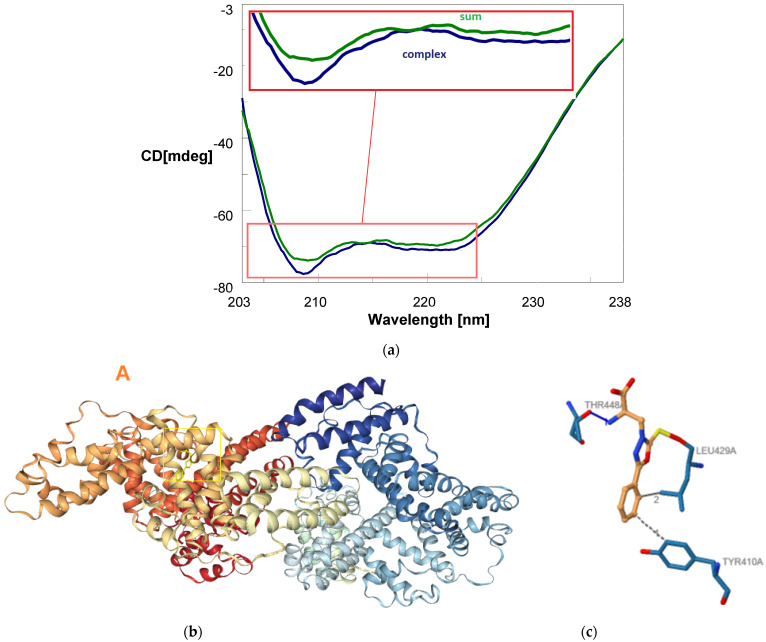
CD binding assay showing complex formation between BSA (0.785 μM) and compound **5** (37.5 nmol) in PBS 1x at pH 7.4 and 22 °C. The blue curve displays the CD spectrum recorded post-mixing of BSA and compound **5** solutions in a tandem dual chamber quartz cell, while the green curve represents the CD spectrum before mixing the two solutions (**a**). 3D pose view depicting the top-ranked pose predicted by HDOCK software for the molecular docking of BSA with compound **5**. The ligand is represented in yellow color within the BSA protein structure (chain A, as indicated by the orange letter ‘A’) (**b**). 3D diagram illustrating the interaction between BSA and compound **5** for the top-1 pose as visualized by PLIP software. Dotted grey lines represent hydrophobic interactions, while blue lines denote hydrogen bonds. For additional information on the residues involved and other bond characteristics, please refer to Table S1 in the Supporting Information of this work (**c**).

In certain tissue regeneration applications, it is crucial for regenerative therapeutics to effectively reach deep targets within the organism, necessitating transportation through the bloodstream [51]. Therefore, we conducted investigations into the capacity of serum albumins, particularly BSA, as a model to bind compound **5**. This assessment is pivotal for applications requiring efficient transport through the bloodstream to reach intended targets [52]. Thus, we conducted binding assays employing circular dichroism (CD), a technique used to study the secondary structure of proteins, nucleic acids, and other chiral molecules [53,54,55], which demonstrated discernible CD spectral differences upon mixing a solution of BSA with compound **5**, confirming effective binding (Figure 3a and Figure S3). Molecular docking using HDOCK revealed that compound **5** binds to BSA and is hosted within a cavity in chain A of the BSA dimer, forming both hydrophobic interactions and hydrogen bonding (Figure 3b,c, Tables S1 and S2). The impact on specific secondary structures resulting from the interaction of compound **5** with BSA was also examined through CD deconvolution (Table 1). CD spectra deconvolution [56] is a computational method employed to analyze complex CD protein spectra and extract quantitative information about their secondary structure composition. By fitting experimental CD spectra with reference spectra representing known secondary structure elements, deconvolution algorithms can estimate the relative contributions of structures such as α-helix, β-sheet, and random coil within the sample. This process allows researchers to gain insights into the structural changes occurring in biomolecules under different experimental conditions, such as temperature, pH, or ligand binding. CD spectra deconvolution is particularly useful in elucidating structural transitions, assessing protein folding/unfolding processes, and monitoring conformational changes induced by ligand binding or environmental perturbations. In our study, we employed CD spectra deconvolution to explore the binding interaction between BSA and ligand compound **5**. By analyzing the changes in the BSA CD spectra in the presence of compound **5**, we aimed to understand the alterations in the protein’s secondary structure induced by ligand binding and to obtain insights into the binding mechanism and affinity between BSA and compound **5**.

Overall, CD deconvolution revealed variations indicating both subtle and significant effects (Table 1) induced by compound **5** on the BSA secondary structure, highlighting its multifaceted impact. The effect of 5 on BSA secondary structure, as observed through CD deconvolution, demonstrates a complex interaction that determines a simultaneous decrease in α-helix content (−6.9%) and an increase in both β-sheet (+4.7%) and random coil (+2.2%) structures (Table 1). This suggests compound **5** has a propensity to induce conformational changes within BSA, potentially slightly destabilizing α-helix structures while promoting β-sheet formation and increasing structural flexibility. Such multifaceted effects highlight the diverse mechanisms by which the ligand can modulate protein secondary structure in our experiments.

## 4. Discussion

The synthesis of compound **5**, a novel non-natural synthetic amino acid, was successfully achieved through a solution-phase approach involving the chiral NiII complex of Schiff’s base of dehydroalanine (Δ-Ala) with the chiral auxiliary (S)-2-N-(N’-benzylprolyl) aminobenzophenone (NiII-(S)-BPB-Δ-Ala), followed by the addition of substituted 1,3,4-oxadiazole 2. This synthetic route resulted in the isolation of compound **5** with 52% yield and high optical purity, demonstrating the efficiency and robustness of the synthetic protocol. The purification steps ensured the recovery of the chiral auxiliary (S)-BPB, allowing for its reuse in future synthetic endeavors. Furthermore, the crystallization process led to the isolation of compound **5** with a satisfactory yield, underscoring its suitability for further characterization (Figures S1, S2 and S4) and subsequent applications. Investigations into the self-assembly properties of compound **5** revealed its propensity to form supramolecular networks, as evidenced by Dynamic Light Scattering studies. The DLS data suggests a dominant size of approximately 391 nm with a polydispersity index (PdI) of 0.262, indicating a relatively narrow size distribution and suggesting uniformity in the size range of particles within the sample. Computational studies using HDOCK software provided insights into the molecular interactions governing the assembly of compound **5**, elucidating distinct modes of interaction and potential variations in stability and binding affinity within the aggregates. The formation of higher-order aggregates, including a 512-mer model, highlights the capacity of compound **5** to organize into complex structures with therapeutic cargo-hosting capabilities, thus presenting a promising scaffold for tissue regenerative applications. Cellular studies demonstrated the ability of compound **5** to promote fibroblast growth, suggesting its utility in tissue regeneration approaches. Additionally, investigations into the interaction of compound **5** with serum albumins, particularly BSA, provided valuable insights into its potential as a carrier for therapeutic cargo delivery. The CD binding assays and molecular docking studies revealed the effective binding of compound **5** to BSA, with detailed structural insights into the binding mode and resulting conformational changes in BSA secondary structure. The effect of compound **5** on BSA secondary structure, as observed through CD deconvolution, reveals a complex interaction resulting in a simultaneous decrease in α-helix content (−6.9%) and an increase in both β-sheet (+4.7%) and random coil (+2.2%) structures. The observed alterations in BSA secondary structure highlight the diverse mechanisms by which compound **5** can modulate protein interactions encompassing both helical region destabilization and beta-sheet formation.

## 5. Conclusions

In conclusion, this study details the synthesis, purification, and characterization of a novel non-natural synthetic amino acid. Starting from the chiral NiII complex of Schiff’s base of dehydroalanine (Δ-Ala) with the chiral auxiliary (S)-2-N-(N’-benzylprolyl) aminobenzophenone, subsequent addition of substituted 1,3,4-oxadiazole yielded the desired α-amino acid. After isolation and recovery of the chiral auxiliary, the final amino acid product was obtained through crystallization. Notably, the self-assembly of the synthetic amino acid demonstrated by DLS revealed nanostructures with an average size of 391 nm, extending to the low micrometric size range, which is promising for its potential application in tissue regenerative therapies. In fact, cellular biological assays suggested the synthetic amino acid’s capability to enhance fibroblast cell growth, underscoring its potential utility in tissue regenerative applications. Circular dichroism spectroscopy provided insights into its interaction with serum albumins, with BSA used as a model protein. The demonstrated binding of compound **5** to serum albumin is a valuable property, as this interaction potentially facilitates the effective transportation of 5 in the bloodstream. Additionally, CD deconvolution analysis shed light on the changes in secondary structures of BSA upon interaction with the amino acid ligand. Moreover, molecular docking studies using HDOCK software elucidated the probable binding mode of the ligand within the BSA structure, providing valuable insights into its molecular interactions. In silico oligomerization experiments yielded a model of high-order aggregates, including a 512-mer nanostructure, which exhibited a cavity capable of hosting therapeutic cargoes and biomolecules. This suggests the potential of the synthetic amino acid as a scaffold for cell adhesion and growth in tissue regenerative applications. Overall, the findings of this study underscore the potential of synthetic amino acids for tissue regenerative therapeutics. The insights gained into its molecular interactions and aggregation behavior further contribute to our understanding of its potential applications in tissue engineering and regenerative medicine.

## Figures and Tables

**Table 1:** Effect of Compound **5** on BSA Secondary Structure.

Secondary Structure	Δ(x-BSA) ^1^ (%)	Change (%)
α-helix	62.2–69.1	−6.9
β-sheet	10.5–5.8	+4.7
Random coil	27.3–25.1	+2.2

^1^ Δ(x-BSA) (%) represents the change in secondary structure content relative to BSA induced by 5.

## Data Availability

The data presented in the study are included in the article and in the Supplementary Material, further inquiries can be directed to the corresponding author.

## Supplementary Materials

The following supporting information can be downloaded at: https://www.mdpi.com/article/10.3390/biom14050579/s1, Figure S1: ^1^H NMR spectral analysis of amino acid 5; Figure S2: ^13^C NMR spectral analysis of amino acid 5; Figure S3: Three-dimensional view of the complex of 5 with BSA for the model 1 corresponding to the top-1 pose. Notice the variation in docking scores, which range from −117.52 to −140.91, with the top-1 pose corresponding to the model with the best HDOCK score (−140.91); Figure S4: IR and ESI MS analysis of compound **5**. Table S1: Interface residues (type and number) and distances from the ligand for both A and B chains of BSA for the top-1, top-2, and top-3 poses. Notice how comp 1 binds chain A at the level of the subdomain IIIA; Table S2: Interaction between the target protein and compound **5** for the top-1 pose as visualized by PLIP software.
