# Supplementary material for: BSA Binding and Aggregate Formation of a Synthetic Amino Acid with Potential for Promoting Fibroblast Proliferation: An In Silico, CD Spectroscopic, DLS, and Cellular Study
Source: Biomolecules. 2024 May 14;14(5):579. doi: 10.3390/biom14050579 (PMC11118884; doi:10.3390/biom14050579)
Supplement: Supplementary file 1 [file biomolecules-14-00579-s001.zip › biomolecules-2962699-supplementary.pdf]

biomolecules

MDPI

Supplementary Information

BSA binding and aggregate formation of a synthetic amino acid with potential for promoting fibroblast proliferation: an in silico, CD spectroscopic, DLS, and cellular study

Hayarpi Simonyan[1#], Rosanna Palumbo[2#], Satenik Petrosyan[1], Anna Mkrtchyan[1], Armen Galstyan[3], Ashot Saghyan[1], Pasqualina Liana Scognamiglio[4], Caterina Vicidomini[2], Marta Fik-Jaskolka[5], Giovanni N. Roviello[2*]

[1] Institute of Pharmacy, Yerevan State University, 1 Alex Manoogian Str., Yerevan 0025, Armenia
[2] Institute of Biostructures and Bioimaging, Italian National Council for Research (IBB-CNR), Area di Ricerca Site and Headquarters, Via Pietro Castellino 111, 80131 Naples, Italy
[3] Department of Chemistry, Yerevan State University, 1 Alex Manoogian Str., Yerevan 0025, Armenia
[4] Department of Sciences, University of Basilicata, Via dell'Ateneo Lucano 10, 85100 Potenza, Italy
[5] Faculty of Chemistry, Adam Mickiewicz University in Poznań, Uniwersytetu Poznańskiego 8, 61-614 Poznań, Poland

* Correspondence: giovanni.roviello@cnr.it; +390812203415
[#]these authors contributed equally to this work

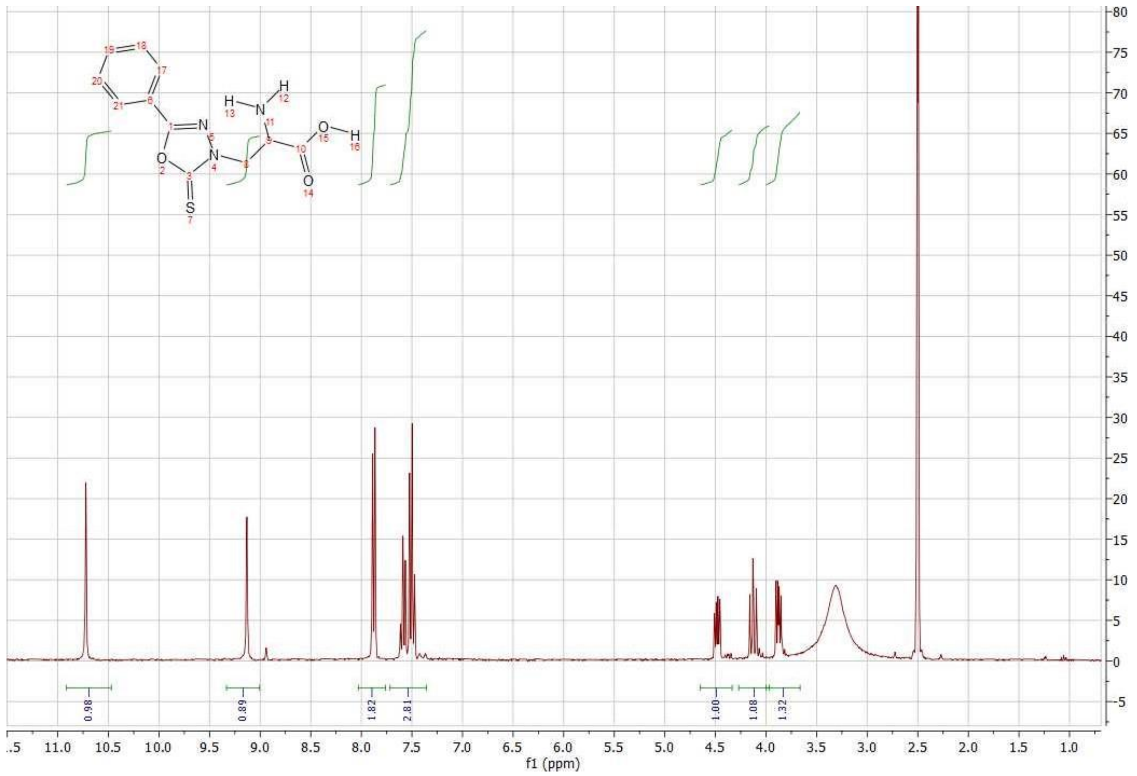

Figure S1. ^{1}H NMR spectral analysis of amino acid 5

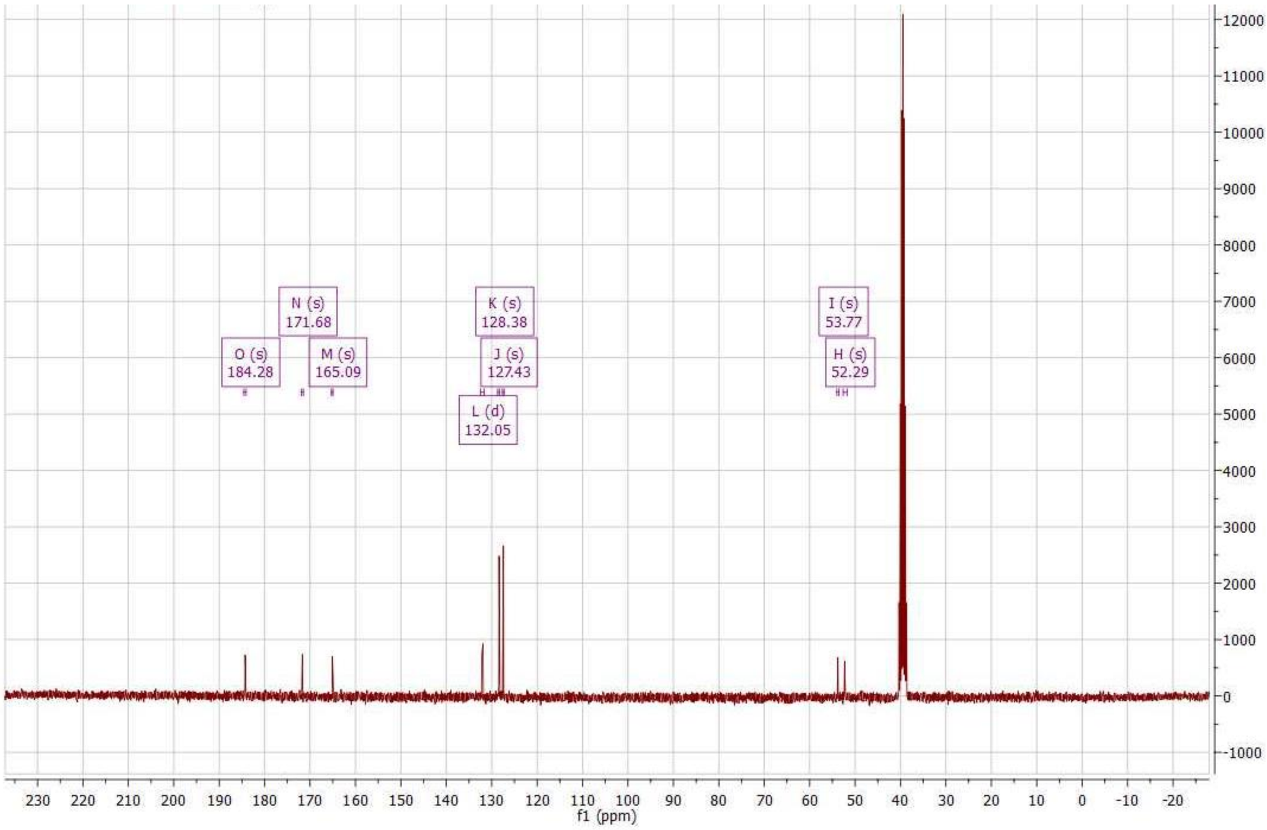

Figure S2. ^{13}C NMR spectral analysisi of amino acid 5

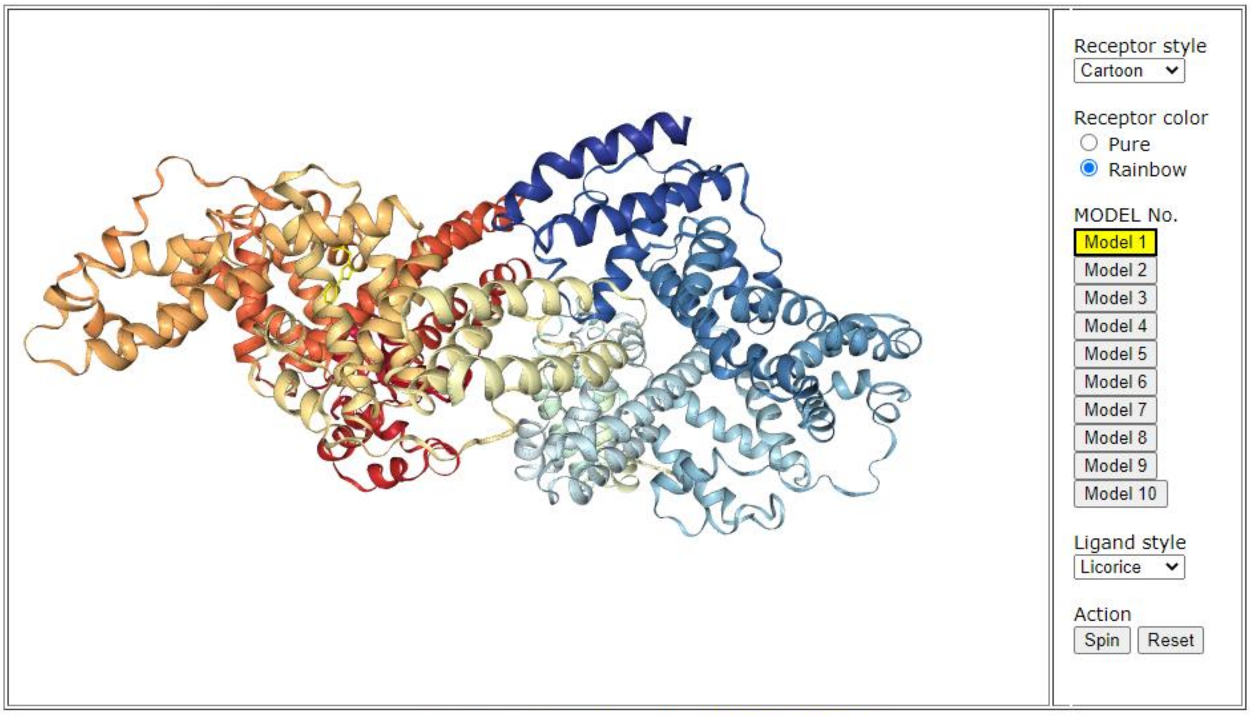

Complex Template Information (Click to Show)

Summary of the Top 10 Models

**Rank**	**1**	**2**	**3**	**4**	**5**	**6**	**7**	**8**	**9**	**10**
**Docking Score**	-140.91	-137.59	-135.39	-131.18	-130.79	-125.06	-121.99	-118.09	-118.06	-117.52

Figure S3. Three-dimensional view of the complex of 5 with BSA for the model 1 corresponding to the top-1 pose. Notice the variation in docking scores, which range from -117.52 to -140.91, with the top-1 pose corresponding to the model with the best HDOCK score (-140.91).

Table S1: Interface residues (type and number) and distances from the ligand for both A and B chain of BSA for the top-1, top-2 and top-3 poses. Notice how comp 1 binds chain A at level of the subdomain IIIA

LEU	386A	4.099	-140.91
ILE	387A	3.834	
ASN	390A	3.293	
CYS	391A	1.865	
PHE	394A	4.986	
PHE	402A	3.559	
LEU	406A	4.290	
ARG	409A	3.910	
TYR	410A	3.203	
SER	428A	4.653	
LEU	429A	1.853	
GLY	430A	4.084	
LYS	431A	4.398	

VAL	432A	2.064	
GLY	433A	2.931	
THR	434A	4.821	
CYS	436A	4.096	
CYS	437A	1.635	
THR	448A	2.959	
LEU	452A	3.823	
SER	488A	3.322	
LEU	386B	3.710	-137.59
ILE	387B	3.883	
ASN	390B	2.817	
CYS	391B	1.995	
PHE	394B	4.663	
PHE	402B	3.100	
LEU	406B	4.594	
ARG	409B	3.469	
TYR	410B	3.429	
LYS	413B	4.247	
SER	428B	4.543	
LEU	429B	1.804	
GLY	430B	4.037	
LYS	431B	4.743	
VAL	432B	2.169	
GLY	433B	2.868	
THR	434B	4.932	
CYS	436B	4.635	
CYS	437B	1.864	
THR	448B	3.933	
LEU	452B	3.249	
SER	488B	3.451	
PHE	501A	3.070	-135.39
PHE	506A	3.342	
PHE	508A	3.893	
ALA	527A	4.141	
LEU	528A	4.859	
LEU	531A	3.376	
HIS	534A	2.670	
LYS	535A	2.751	
VAL	546A	4.352	

PHE	550A	3.254
LEU	574A	3.572
VAL	575A	1.908
VAL	576A	4.774
THR	578A	3.031
GLN	579A	2.910
LEU	582A	4.388

Table S2: Interaction between the target protein and compound 5 for the top-1 pose as visualized by PLIP software

Hydrophobic Interactions ····

Index	Residue	AA	Distance	Ligand Atom	Protein Atom
1	410A	TYR	3.40	9322	3305
2	429A	LEU	3.99	9321	3454

Hydrogen Bonds —

Index	Residue	AA	Distance H-A	Distance D-A	Donor Angle	Protein donor?	Side chain	Donor Atom	Acceptor Atom
1	448A	THR	2.17	2.96	132.56	×	✔	9309 [N3]	3593 [O3]

···· *Hydrophobic Interaction*
— *Hydrogen Bond*

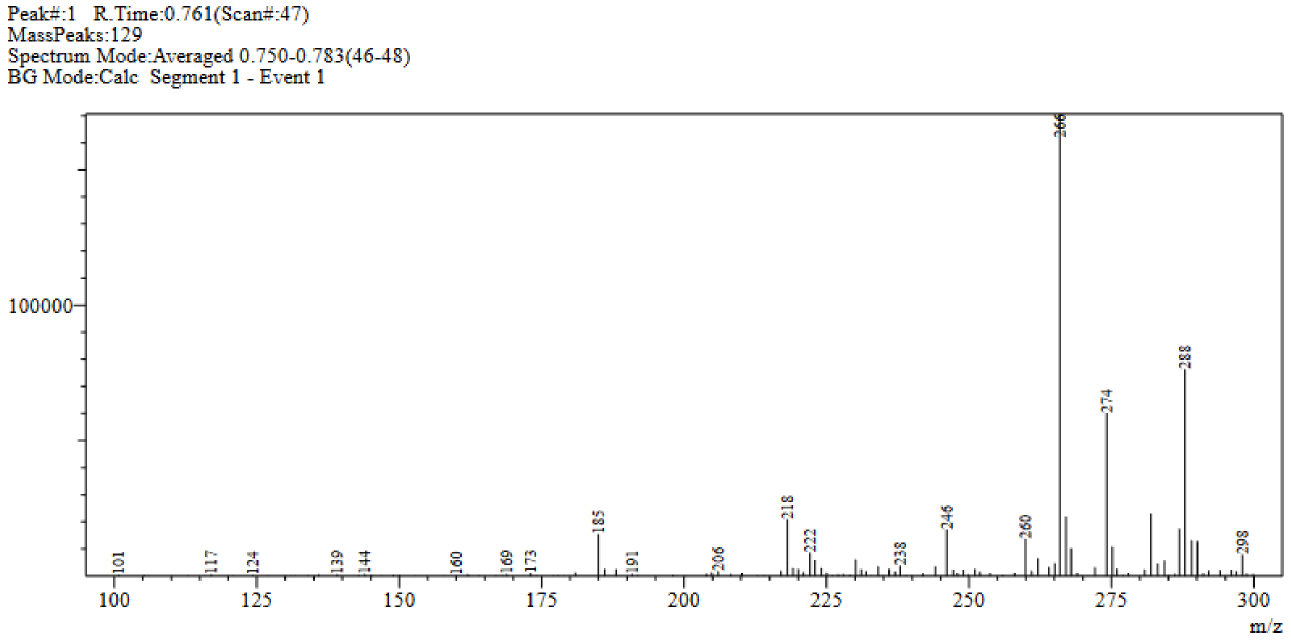

MASS Peak Table TIC

Peak#	Ret. Time	Area	Base Peak m/z
1	0.761	11187839	265.95
Total		11187839	

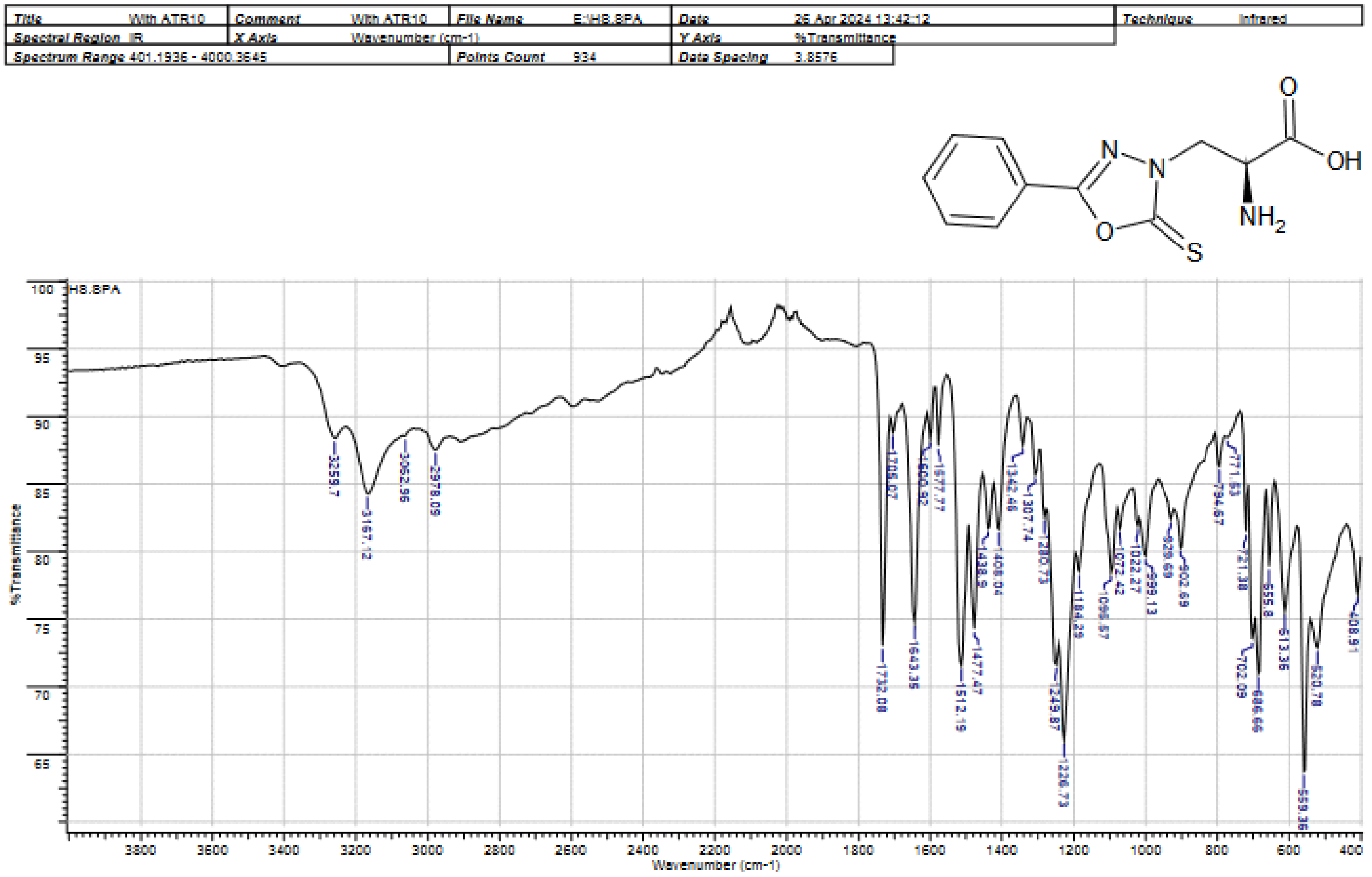

Figure S4. ESI MS (up) and IR (bottom) analysis of compound 5
